# Sociodemographic Factors, Comorbidities, In-Hospital Complications, and Outcomes of Ischaemic Stroke Patients in the Context of the COVID-19 Pandemic in Lithuania: A Retrospective, Record-Based, Single-Centre Study

**DOI:** 10.7759/cureus.45553

**Published:** 2023-09-19

**Authors:** Erika Jasukaitienė, Lolita Šileikienė, Šarūnas Augustis, Abdonas Tamošiūnas, Dalia Lukšienė, Daina Krančiukaitė-Butylkinienė, Gintarė Šakalytė, Diana Žaliaduonytė, Ričardas Radišauskas

**Affiliations:** 1 Department of Population Studies, Institute of Cardiology, Medical Academy, Lithuanian University of Health Sciences, Kaunas, LTU; 2 Institute of Cardiology, Medical Academy, Lithuanian University of Health Sciences, Kaunas, LTU; 3 Department of Cardiology, Medical Academy, Lithuanian University of Health Sciences, Kaunas, LTU

**Keywords:** ischaemic stroke, sex, comorbidities, in-hospital complications, covid-19, outcomes, risk

## Abstract

Introduction: The role of COVID-19 regarding in-hospital complications and poor outcomes for patients with ischaemic stroke (IS) is still important to explore. The aim of this study was to evaluate the risk of in-hospital lethality for IS patients respectively to their comorbidities and in-hospital complications in the context of the COVID-19 pandemic.

Methods: We identified 1898 acute IS patients (749 men and 1149 women) admitted to the Lithuanian University of Health Sciences Kaunas Hospital, Lithuania, from December 2020 to February 2022. The sociodemographic, clinical, and outcome features of the patients were evaluated deploying appropriate statistical tests. Hazard ratios and 95% confidence intervals were estimated by the Cox proportional hazards regression for hospital lethality.

Results: The risk of in-hospital lethality was 2.22 times higher in men suffering from IS and chronic ischaemic heart disease (cIHD) compared to those with IS and isolated arterial hypertension (iAH) (p < 0.05). COVID-19 elevated the risk of in-hospital lethality in men by 3.16 times (p < 0.05). In comorbid women with type two diabetes mellitus (DM) or cIHD, the risk of in-hospital lethality was two times higher compared to those with iAH (p < 0.05). The risk of in-hospital lethality increased significantly in both men and women, with the total number of in-hospital complications increasing per one unit.

Conclusions: Of the comorbidities studied, DM and cIHD together with COVID-19 elevated the risk of in-hospital lethality significantly. Within the acute in-hospital complications, pneumonia with respiratory failure and acute renal failure showed the most significant prognostic value anticipating lethal outcomes for IS patients.

## Introduction

Evidence from the studies published during the second decade of the 21st century indicates that the annual number of strokes and deaths due to stroke increased [1]. It is estimated that the number of people having a stroke and living with the long-term effects of stroke will rise in the coming decades, causing a burden for healthcare systems and populations [2]. Stroke is the second most common single cause of death in Europe [3]. While the number of people living with stroke is estimated to increase by 27% between 2017 and 2047 in the European Union, the largest increase in incidence and mortality is projected for Lithuania [2].

The fastest-growing risk factor for stroke between 1990 and 2019 was a high body mass index, and the coexistence of other risk factors was associated with higher risks of ischaemic heart disease (IHD) [1].

The overall in-hospital mortality for stroke was estimated at 9.3%; as for cardiovascular diseases (CVD), females spent 12% longer in hospital than males [3]. There were ongoing efforts to find indicators to predict clinical outcomes in patients with stroke [4].

In our earlier study performed in Kaunas, Lithuania among the population aged 25 to 64 years who experienced a first-ever stroke between 1986 and 2011, most of all stroke patients suffered from ischaemic stroke (IS). IS was significantly more frequent in males than in females and long-term survival was better in women than men [5].

Since the beginning of the pandemic, COVID-19 affected over 673 million people worldwide, causing more than 6.7 million deaths [6]. In Lithuania (with more than 2.6 million population), there have been around 1.3 million estimated cases of COVID-19 with more than 9,500 deaths until now [6].

New evidence on the effects of COVID-19 disease and vaccines against COVID-19 is important to better understand the potential risks they pose to the course and outcome of CVD and stroke. Stroke requires immediate healthcare response, which has been quite challenging during the lockdowns. The effect of COVID-19, as comorbidity, on complications and stroke outcomes, and long-term consequences for health needs to be evaluated.

COVID-19 is not just a respiratory disease and can affect other organs, including the brain [7]. Several studies have linked exposure to COVID-19 with a higher incidence of acute IS [8,9]. Although respiratory symptoms are most reported, neurological symptoms are increasingly recognized and are registered in every second person [10,11], with a significant risk of acute IS due to such SARS-CoV-2-related pathophysiological mechanisms, like hypercoagulability, general inflammation, vasculopathy, and others [11,12]. IS was more predominant than haemorrhagic stroke in COVID-19 patients who experienced a stroke [13]. Emerging cohort studies suggest a potential increased risk for vascular diseases in patients with COVID-19, especially for those experiencing both COVID-19 and stroke [14].

It is important to understand whether the augmented cardiovascular risk is associated with COVID-19, including acute IS [7,9]. Around one-third of patients were found to have at least one comorbidity, with arterial hypertension (AH), diabetes mellitus (DM), CVD, and malignant tumours being most often described [15]. Similarly, little is known about any specific characteristics of COVID-19-associated stroke.

The aim of this study was to evaluate the risk of in-hospital lethality for IS patients according to comorbidities, and in-hospital complications in the context of the COVID-19 pandemic.

## Materials and methods

This is a retrospective, record-based, observational descriptive study regarding epidemiological and clinical features of acute IS patients, admitted to the Kaunas Hospital of the Lithuanian University of Health Sciences (LUHS), Lithuania, from December 2020 to February 2022.

Patients and sample size

The sample size consisted of IS patients (749 men and 1149 women) to whom interventional treatment of IS was contraindicated according to the decisions at the tertiary stroke centre.

Inclusion criteria included patients with IS, as defined by the WHO [16]; cases of recurrent IS within the period from December 2020 to February 2022 were included, but each person with a stroke was included only as one, i.e., the earliest case. Patients with a transient ischaemic attack (TIA) or a silent brain infarction (as suspected from CT) were excluded.

Data regarding sociodemographic, clinical, and outcome factors were obtained from patients’ medical records. The outcome was defined in terms of demographics, comorbidity, in-hospital complications, and lethal outcomes.

Patients with suspected stroke were first referred to the tertiary stroke centre, where CT was instantly performed confirming the IS diagnosis. The indications and contraindications for intravenous thrombolysis and thrombectomy were thoroughly assessed. The patients in our study cohort had absolute contraindications against the interventional IS treatment, including >4.5 hours from onset of symptoms, previous intracerebral bleeding, intracranial or spinal surgery in the previous three months, intracerebral neoplasm, infective endocarditis, aortic arch dissection, severe and uncontrolled arterial hypertension, active internal bleeding, and others. The patients were relocated to Kaunas Hospital of the LUHS for non-interventional IS treatment.

Definition of comorbidity and in-hospital complications

Recurrent and chronic health problems were accepted as comorbid main cerebrovascular risk factors. The study patients were grouped according to the International Statistical Classification of Diseases and Related Health Problems, Tenth Revision, Australian Modification (ICD-10-AM) as follows: (1) having isolated AH (I11), (2) having type 2 DM (E11), and (3) having complicated chronic IHD (I25) with heart failure (I50) or atrial fibrillation and flutter (I48), or angina pectoris (I20). The comorbidities of these three patient groups were diagnosed before admission to the stroke hospital and all the patients were already receiving appropriate treatment.

Moreover, many study patients suffered from multiple comorbid diseases at once, such as chronic tubulointerstitial nephritis (N11), disorders of fluid, electrolyte, and acid-base balance (E87), chronic kidney disease (N18), vascular dementia (F01), and Parkinson's disease (G20).

Pneumonia (J18), pulmonary embolism (I26), respiratory failure (J96), acute tubulointerstitial nephritis (N10), and acute renal failure (N17) were identified as important acute in-hospital complications together with pre-existing comorbidities.

Statistical analysis

Descriptive characteristics (prevalence rates, means, and standard errors (SE)) were calculated for variables separately for men and women. The differences in means of variables between the sex groups and between the IS groups were assessed using T-test and ANOVA with Bonferroni multiple comparison tests. A chi-squared test and z-test with Bonferroni corrections were used to assess the differences in categorical variables. P-values < 0.05 were considered statistically significant.

Hazard ratios (HR) and 95% confidence intervals (CI) were estimated by the Cox proportional hazards regression for hospital lethality. Two models were assessed. Model 1 adjusted for age. Model 2 adjusted for age, COVID-19 status, IS group + comorbidities (IS + AH; IS + DM; IS + chronic IHD), and five different in-hospital complications. Also, HR was estimated by the Cox proportional hazards regression for hospital lethality according to the number of in-hospital complications, age, COVID-19 status, and IS group + comorbidities (IS + AH; IS + DM; IS + chronic IHD).

Statistical analyses were performed using IBM SPSS Statistics version 27.0 (IBM Corp., Armonk, NY).

## Results

Table 1 shows the baseline characteristics of IS patients admitted to Kaunas Hospital of LUHS differentiated by sex; 60.5% of IS patients were women and 39.5% were men. The mean age was significantly higher in women compared to men. The prevalence of specific comorbidities, such as heart failure, atrial fibrillation and flutter, chronic tubulointerstitial nephritis, and other disorders of fluid, electrolyte, and acid-base balance, was significantly higher in women compared to men. The rate of in-hospital complication, acute tubulointerstitial nephritis, was also significantly higher in the group of women compared to the group of men. The proportion of deceased among IS patients was significantly higher in women compared to men (19.7% and 15.0%, respectively; p < 0.05). The proportions of COVID-19 diagnosis, the status of vaccination for COVID-19, and the number of vaccinations in men and women groups did not differ.

**Table 1:** Baseline characteristics of ischaemic stroke patients admitted to Kaunas Hospital of the Lithuanian University of Health Sciences from December 2020 to February 2022. ^& ^Prior to hospitalization; * p < 0.05; ** p < 0.01; *** p < 0.001 compared to men group.

Variables	Men	Women	Total
Sex, N (%)	749 (39.5)	1149 (60.5)	1898 (100.0)
Age, years, mean (SD)	72.2 (11.0)	79.8 (9.5)***	76.8 (10.8)
Age group, years (%)			
25-64	26.2	7.3*	14.8
65-84	61.4	61.7	61.5
85+	12.4	31.0*	23.7
Specific comorbidity, N (%)			
COVID-19 (U07)	83 (11.1)	99 (8.6)	182 (9.6)
Hypertensive heart disease (I11)	642 (85.7)	954 (83.0)	1596 (84.1)
Angina pectoris (I20)	33 (4.4)	75 (6.5)	108 (5.7)
Heart failure (I50)	47 (6.3)	109 (9.5)*	156 (8.2)
Chronic ischaemic heart disease (I25)	41 (5.5)	63 (5.5)	104 (5.5)
Atrial fibrillation and flutter (I48)	229 (30.6)	488 (42.5)***	717 (37.8)
Sequelae of cerebrovascular disease (I69)	38 (5.1)	63 (5.5)	101 (5.3)
Chronic tubulointerstitial nephritis (N11)	10 (1.3)	38 (3.3)**	48 (2.5)
Chronic kidney disease (N18)	23 (3.1)	35 (3.0)	58 (3.1)
Other disorders of fluid, electrolyte, and acid-base balance (E87)	25 (3.3)	62 (5.4)*	87 (4.6)
Vascular dementia (F01)	12 (1.6)	33 (2.9)	45 (2.4)
Parkinson's disease (G20)	12 (1.6)	18 (1.6)	30 (1.6)
Other disorders of brain (G93)	15 (2.0)	28 (2.4)	43 (2.3)
Number of comorbidities, mean (SD)	1.6 (0.9)	1.8 (1.0)	1.7 (1.0)
Number of comorbidities, N (%)			
-2	641 (85.6)	929 (80.9)*	1570 (82.7)
3-4	101 (13.5)	201 (17.5)*	302 (15.9)
5+	7 (0.9)	19 (1.7)	26 (1.4)
In-hospital complications, N (%)			
Pneumonia, organism unspecified (J18)	67 (8.9)	98 (8.5)	165 (8.7)
Pulmonary embolism (I26)	9 (1.2)	9 (0.8)	18 (0.9)
Respiratory failure, not elsewhere classified (J96)	31 (4.1)	60 (5.2)	91 (4.8)
Acute tubulointerstitial nephritis (N10)	34 (4.5)	103 (9.0)***	137 (7.2)
Acute renal failure (N17)	9 (1.2)	21 (1.8)	30 (1.6)
Number of in-hospital complications, mean (SD)	0.2 (0.5)	0.3 (0.6)***	0.2 (0.5)
Number of patients with at least one in-hospital complication, N (%)	125 (16.7)	232 (20.2)	357 (18.8)
Length of hospitalization, mean (SD)	16.7 (18.9)	18.1 (19.3)*	17.6 (19.1)
Length of hospitalization, median	10.0	11.0	10.0
Vaccination for COVID-19			
Yes^&^	61 (8.1)	81 (7.0)	142 (7.5)
No	688 (91.9)	1068 (93.0)	1756 (92.5)
Number of vaccinations, N (%)			
1	6 (9.8)	7 (8.6)	13 (9.2)
2 and more	55 (90.2)	74 (91.4)	129 (90.8)
Deceased, N (%)	112 (15.0)	226 (19.7)*	338 (17.8)
Time to death, days, mean (SD)	11.5 (16.2)	16.3 (27.9)	14.7 (24.7)
Survivors, N (%)	637 (85.0)	923 (80.3)*	1560 (82.2)

Sociodemographic factors, specific comorbidities, in-hospital complications, COVID-19 vaccination, and outcomes according to IS patients’ groups are presented in Table 2. At first, we compared the distribution and means of variables presented in this table, according to gender in each IS group. The mean age in all three groups was significantly higher in women compared to men. However, the proportion of men in the youngest age group (25-64 years) was significantly higher compared to women in all three IS groups. After the evaluation of the frequency of in-hospital complications in all three IS groups, only the proportion of acute tubulointerstitial nephritis was significantly higher in women compared to men in group 1 and group 3. The proportions of vaccination for COVID-19 in group 3 were significantly higher in men compared to women. The mean time to death (in days) in group 3 was significantly longer in women compared to men.

**Table 2:** Sociodemographic factors, specific comorbidities, in-hospital complications, COVID-19 vaccination, and outcomes according to ischaemic stroke patients’ groups. ^& ^Prior to hospitalization; * p < 0.05 compared to men group; ^a ^p < 0.05 compared to group 1 in men and women groups; ^b ^p < 0.05 compared to group 2 in men and women groups. The proportions of comorbidity codes (ICD-10 codes I11, I20, I50, I25, I48, E87, F01, G20, and G93) were not compared between groups because these codes were included in one of groups 1, 2, or 3. IS: ischaemic stroke; AH: arterial hypertension; DM: diabetes mellitus; IHD: ischaemic heart disease; ICD: International Classification of Diseases.

Variables	Group 1		Group 2		Group 3	
	IS + AH		IS + DM		IS + chronic IHD	
	Men	Women	Men	Women	Men	Women
	N = 330	N = 358	N = 85	N = 138	N = 334	N= 653
Age, years, mean (SD)	69.3 (10.4)	78.1 (10.7)*	70.7 (9.1)	76.8 (8.8)*	75.6 (11.1)^a, b^	81.3 (8.6)*^, a, b^
Age group, years, N (%)						
25-64	115 (34.8)	40 (11.2)*	23 (27.1)	14 (10.1)*	58 (17.4)^a, b^	30 (4.6)*^, a^
65-84	194 (58.8)	220 (61.4)*	58 (68.2)	101 (73.2)*^, a^	208 (62.2)	388 (59.4)^b^
85+	21 (6.4)	98 (27.4)*	4 (4.7)	23 (16.7)*^, a^	68 (20.4)^a, b^	235 (36.0)*^, a, b^
Specific comorbidity, N (%)						
COVID-19 (U07)	31 (9.4)	27 (7.5)	11 (12.9)	10 (7.2)	41 (12.3)	62 (9.5)
Hypertensive heart disease (I11)	330 (100.0)	358 (100.0)	71 (83.5)	106 (76.8)	241 (72.2)	490 (75.0)
Angina pectoris (I20)	0 (0.0)	0 (0.0)	0 (0.0)	1 (0.7)	33 (9.9)	74 (11.3)
Heart failure (I50)	0 (0.0)	0 (0.0)	3 (3.5)	4 (2.9)	44 (13.2)	105 (16.1)
Chronic IHD (I25)	0 (0.0)	0 (0.0)	0 (0.0)	1 (0.7)	41 (12.3)	62 (9.5)
Atrial fibrillation and flutter (I48)	0 (0.0)	0 (0.0)	4 (4.7)	13 (9.4)	225 (67.4)	475 (72.7)
Sequelae of cerebrovascular disease (I69)	15 (4.5)	13 (3.6)	1 (1.2)	8 (5.8)	22 (6.6)	42 (6.4)
Chronic tubulointerstitial nephritis (N11)	2 (0.6)	8 (2.2)	0 (0.0)	4 (2.9)	8 (2.4)	26 (4.0)
Chronic kidney disease (N18)	3 (0.9)	4 (1.1)	4 (4.7)^a^	2 (1.4)	16 (4.8)^a^	29 (4.4)^a^
Other disorders of fluid, electrolyte, and acid-base balance (E87)	0 (0.0)	0 (0.0)	2 (2.4)	6 (4.3)	23 (6.9)	56 (8.6)
Vascular dementia (F01)	0 (0.0)	0 (0.0)	0 (0.0)	2 (1.4)	12 (3.6)	31 (4.7)
Parkinson's disease (G20)	0 (0.0)	0 (0.0)	0 (0.0)	0 (0.0)	12 (3.6)	18 (2.8)
Other disorders of brain (G93)	0 (0.0)	0 (0.0)	1 (1.2)	4 (2.9)	14 (4.2)	24 (3.7)
Number of comorbidities, mean (SD)	1.1 (0.3)	1.1 (0.4)	1.1 (0.7)	1.2 (0.6)	2.2 (1.0)	2.3 (1.0)
Number of comorbidities, N (%)						
0-2	329 (99.7)	356 (99.4)	83 (97.6)	134 (97.1)	234 (70.1)	446 (68.3)
3-4	1 (0.3)	2 (0.6)	2 (2.4)	4 (2.9)	93 (27.8)	191 (29.2)
5+	0 (0.0)	0 (0.0)	0 (0.0)	0 (0.0)	7 (2.1)	16 (2.5)
In-hospital complications, N (%)						
Pneumonia, organism unspecified (J18)	19 (5.8)	15 (4.2)	4 (4.7)	6 (4.3)	44 (13.2)^a^	77 (11.8)^a, b^
Pulmonary embolism (I26)	2 (0.6)	2 (0.6)	1 (1.2)	2 (1.4)	6 (1.8)	5 (0.8)
Respiratory failure, not elsewhere classified (J96)	4 (1.2)	9 (2.5)	4 (4.7)	8 (5.8)	23 (6.9)^a^	43 (6.6)^a^
Acute tubulointerstitial nephritis (N10)	6 (1.8)	20 (5.6)*	5 (5.9)	10 (7.2)	3 (6.9)^a^	73 (11.2)*^, a^
Acute renal failure (N17)	2 (0.6)	2 (0.6)	0 (0.0)	1 (0.7)	7 (2.1)	18 (2.8)^a^
Number of in-hospital complications, mean (SD)	0.1 (0.3)	0.1 (0.4)	0.2 (0.5)	0.2 (0.4)	0.3 (0.6)^a, b^	0.3 (0.6)^a, b^
Number of patients with at least one in-hospital complication, N (%)	30 (9.1)	41 (11.5)	11 (12.9)	25 (18.1)	84 (25.1)^a, b^	166 (25.4)^a^
Length of hospitalization, mean (SD)	15.9 (14.2)	17.8 (19.3)	17.9 (12.9)	17.1 (19.3)	17.3 (23.7)	18.5 (19.2)
Length of hospitalization, median	10.0	10.0	13.0	9.0*	10.0	11.0*
Vaccination for COVID-19						
Yes^&^	29 (8.8)	38 (10.6)	10 (11.8)	11 (8.0)	39 (11.7)	46 (7.0)*
Number of vaccinations, N (%)						
1	6 (1.8)	5 (1.4)	0 (0.0)	1 (0.7)	3 (0.9)	5 (0.7)
2 and more	23 (7.0)	33 (9.2)	10 (11.8)	10 (7.3)	36 (10.8)	46 (6.3)
Deceased, N (%)	26 (7.9)	39 (10.9)	11 (12.9)	29 (21.0)^a^	75 (22.5)^a^	158 (24.2)^a^
Time to death, days, mean (SD)	12 (12.1)	17.8 (33.3)	17.4 (17.9)	15.9 (30.8)	10.5 (17.2)	16.0 (26.0)*
Survivors, N (%)	304 (92.1)	319 (89.1)	74 (87.1)	109 (79.0)^a^	259 (77.5)^a^	495 (75.8)^a^

Secondly, we compared the distribution and means of variables presented in Table 2 in IS groups (for men and women separately). The mean age of men and women was highest in group 3 compared to group 1 and group 2. After the evaluation of the frequency of specific comorbidities, only the proportion of chronic kidney disease was significantly higher in men and women from group 3 compared to patients from group 1. After the evaluation of the frequency of in-hospital complications, the proportions of pneumonia, respiratory failure, acute tubulointerstitial nephritis, and acute renal failure were higher in men and women from group 3 compared to men and women in group 1. Also, the total number of in-hospital complications was significantly higher in group 3 compared to group 1. The proportion of deceased among IS patients was significantly higher in men and women from group 3 compared to patients from group 1 (p < 0.05).

The risk of in-hospital lethality for IS plus comorbidities, in-hospital complications, and COVID-19 status according to gender is presented in Table 3. In men, after adjustment for age (Model 1), the risk of in-hospital lethality was 2.22 times higher in group 3 (IS + chronic IHD) compared to group 1 (IS + AH). The risk of in-hospital lethality in men with the diagnosis of COVID-19 was 3.16 times higher compared to men without such a diagnosis. After the evaluation of the prognostic value of in-hospital complications in the men group, we determined that the risk of in-hospital lethality was significantly higher in patients with pneumonia, pulmonary embolism, respiratory failure, and acute renal failure compared with men without these complications. It is important to notice that out of 83 men having COVID-19, 11 men suffered from pneumonia and two men had pneumonia with acute respiratory failure. After an additional adjustment for all variables included in the Cox regression model (Model 2), only COVID-19 diagnosis and in-hospital complications (pneumonia, respiratory failure, and acute renal failure) significantly increased the risk of in-hospital lethality in the group of men.

**Table 3:** Risk of hospital lethality for ischaemic stroke plus comorbidities, in-hospital complications, and COVID-19 status according to gender. Model 1 adjusted for age. Model 2 adjusted for age, COVID-19 status, ischaemic stroke + comorbidities (IS + AH; IS + DM; IS + chronic IHD), and in-hospital complications. * indicates significance. IS: ischaemic stroke; AH: arterial hypertension; DM: diabetes mellitus; IHD: ischaemic heart disease.

Variables		Men	n = 749			Women	n = 1149	
	Model 1		Model 2		Model 1		Model 2	
	HR	95% CI	HR	95% CI	HR	95% CI	HR	95% CI
Age			1.03	1.01-1.05			1.04	1.03-1.06
Ischaemic stroke (IS) + comorbidities								
Group 1 (IS + AH)	1		1		1		1	
Group 2 (IS + DM)	1.45	0.72-2.93	1.38	0.68-2.81	2.21*	1.36-3.59	2.14*	1.31-3.49
Group 3 (IS + chronic IHD)	2.22*	1.40-3.54	1.46	0.90-2.38	2.01*	1.41-2.87	1.63*	1.13-2.34
COVID-19 status (Yes vs. No)	3.16*	1.86-5.39	1.81*	1.02-3.23	1.29	0.76-2.19	1.10	0.65-1.89
In-hospital complications (Yes vs. No)								
Pneumonia, organism unspecified (J18)	5.14*	3.43-7.68	4.12*	2.71-6.27	2.71*	1.98-3.72	1.85*	1.30-2.63
Pulmonary embolism (I26)	2.68*	1.09-6.60	1.44	0.56-3.69	2.39	0.89-6.44	1.70	0.59-4.97
Respiratory failure, not elsewhere classified (J96)	8.69*	5.54-13.6	6.14*	3.79-9.96	6.30*	4.56-8.70	4.73*	3.25-6.86
Acute tubulointerstitial nephritis (N10)	1.44	0.74-2.78	1.12	0.56-2.28	1.68*	1.18-2.40	1.11	0.76-1.63
Acute renal failure (N17)	11.8*	5.90-23.6	4.96*	2.42-10.2	4.33*	2.55-7.34	1.12	0.60-2.12

In women, after adjustment for age (Model 1), the risk of in-hospital lethality was two times higher in group 2 (IS + DM) and group 3 (IS + chronic IHD) compared to group 1 (IS + AH). After the evaluation of the prognostic value of in-hospital complications in the women group, we found that the risk of in-hospital lethality was significantly higher in patients with pneumonia, respiratory failure, acute tubulointerstitial nephritis, and acute renal failure compared with the women without these complications. Out of six women suffering from COVID-19 with pneumonia, three women developed acute respiratory failure. After an additional adjustment for all variables included in the Cox regression model (Model 2), the risk of in-hospital lethality was 2.14 times higher in group 2 and 1.63 times higher in group 3 compared to group 1. The in-hospital complications (pneumonia and respiratory failure) also significantly increased the risk of in-hospital lethality in the women group.

Also, HR was estimated by the Cox proportional hazards regression for in-hospital lethality according to the number of in-hospital complications, adjusted by age, COVID-19 status, IS group + comorbidities (IS + AH; IS + DM; IS + chronic IHD). The risk of in-hospital lethality significantly increased in men and women with an increase in the number of in-hospital complications per one unit; in the men group, HR = 3.54 (95% CI = 2.7-4.58), and in the women group, HR = 1.96 (95% CI = 1.71-2.25).

## Discussion

Stroke is classified into two main categories, i.e., ischaemic and haemorrhagic stroke. IS is more prevalent and has somewhat lower mortality than the latter [17]. IS is determined to have some major risk factors, including age, sex, smoking, metabolic syndrome, physical inactivity, and nutrition [18-23]. These risk factors increase susceptibility to ischaemic health issues as well as a variety of pre-existing comorbidities, such as AH, DM, and chronic IHD, which are globally recognized as conventional vascular risk factors.

Although COVID-19 is principally understood as viral pneumonia, with cough, high fever, shortness of breath and loss of taste and smell as its characteristic features, the virus responsible for this illness, SARS-CoV-2, may influence the presentation of IS as well [8]. Considering the relationship between SARS-CoV-2 and stroke risk, several pathophysiological mechanisms like hypercoagulability, general inflammation, and vasculopathy might be in play [11,24]. For elder patients who have comorbid illnesses, changes in the cardiovascular system, including intravascular volume changes in the setting of serious infection with COVID-19, may increase atrial fibrillation and cardioembolic potential, or more directly lead to changes in cerebral perfusion pressures that exceed the autoregulatory capacities [24].

Previous studies showed that prior to the COVID-19 pandemic, in-hospital lethality rates for IS remained between 11% and 15% [25]. Many factors account for this rate of lethality, including post-stroke complications that occur after stroke and lead to outcomes. Our investigation resulted in somewhat higher in-hospital lethality rates, reaching from 15.0% for men to 19.7% for women in IS patients. This should not be surprising, knowing that all the patients from our study cohort had contraindications for the interventional IS treatment (fibrinolytic therapy or mechanical thrombectomy) because of their worse medical conditions and related enhanced risk of iatrogenic disorders.

Our study demonstrated that the proportion of women was significantly higher compared to men among the admitted IS patients. Women were significantly older and suffered from comorbid conditions such as heart failure, atrial fibrillation, chronic pyelonephritis, and electrolyte imbalance significantly more often. For women, the in-hospital course was complicated with acute cystitis and pyelonephritis significantly more frequently, which can be explained by the anatomical differences in the female urinary tract. The in-hospital lethality was significantly higher in women compared to men as well. The age difference (significantly larger number of elderly females than males) and the contribution of unrecognized or poorly controlled risk factors could also explain the observed differences between the sex groups.

As expected, DM and chronic IHD, both being comorbid principal vascular risk factors of IS, together with SARS-CoV-2 infection, elevated the risk of in-hospital lethality significantly. Within the acute in-hospital complications, pneumonia, respiratory failure, and acute renal failure showed the most significant prognostic value in anticipating lethal outcomes for IS patients. These results correspond to the results obtained from Germany and Spain Stroke Register investigations [25-27] before the COVID-19 pandemic, which identified respiratory infections as important risk factors of post-stroke in-hospital death as well. Consistent with some previous studies, the in-hospital mortality rates for patients with COVID-19 experiencing an IS were even higher relative to patients with other infectious respiratory diseases [28]. Recent data from selected articles reviewing the effect of COVID-19 on neurological conditions documented the relationship between pre-existing neurological diseases, showing an increased risk of hospitalization, admission length, worsening of symptoms, and even mortality in COVID-19 patients [29].

Moreover, in our study, for both men and women suffering from IS, the risk of in-hospital lethality was significantly advanced together with the increasing total number of in-hospital complications. Again, this emphasizes the importance of determining pre-existing comorbidities and predicting acute in-hospital complications for stroke survivors to assess the risk of in-hospital lethality.

Strengths and limitations

Considerable sample size and accurate analysis of patients’ medical records are the important strengths of the present study. Nevertheless, there are some limitations, too. Firstly, the assessment of the initial severity of the neurological deficit after the onset of stroke is unavailable in our study. This limitation occurs because the patients of our study cohort had the absolute contraindications against the interventional IS treatment, including >4.5 hours from onset of symptoms, previous intracerebral bleeding, intracranial or spinal surgery in the previous three months, intracerebral neoplasm, infective endocarditis, aortic arch dissection, severe and uncontrolled arterial hypertension, active internal bleeding, and others. Patients were relocated to our centre for non-interventional IS treatment without receiving the National Institutes of Health Stroke Scale (NIHSS) score, which is obligatory only before intravenous thrombolysis or mechanical thrombectomy in Lithuania. Baseline neurological deficit is still highly important for stroke outcomes.

Secondly, the lack of longer-term follow-up makes it hard to evaluate the effect of the complications and comorbidities on post-hospital mortality; still, this issue is to be resolved by future authored studies. Further, some patients were excluded from our study due to missing or inconsistent values regarding COVID-19-specific vaccination. There were no significant differences in the main characteristics between the included and excluded cases though.

## Conclusions

COVID-19 diagnosis and in-hospital complications (pneumonia, respiratory failure, and acute renal failure) significantly increased the risk of in-hospital lethality in the IS men group. The comorbidities (DM and chronic IHD) and in-hospital complications (pneumonia and respiratory failure) significantly increased the risk of in-hospital lethality in the IS women group. The risk of hospital lethality significantly increased in men and women with the increasing total number of in-hospital complications. Results of the present study indicate that the spectrum of pre-existing comorbidities and acute in-hospital complications predict in-hospital lethality in IS survivors, and in the context of the pandemic, further investigations are needed on the possibilities to reduce in-hospital deaths in stroke patients.
